# Nonhemolytic *Listeria monocytogenes*—Prevalence Rate, Reasons Underlying Atypical Phenotype, and Methods for Accurate Hemolysis Assessment

**DOI:** 10.3390/microorganisms10020483

**Published:** 2022-02-21

**Authors:** Iwona Kawacka, Agnieszka Olejnik-Schmidt, Marcin Schmidt

**Affiliations:** Department of Food Biotechnology and Microbiology, Poznan University of Life Sciences, Wojska Polskiego 48, 60-627 Poznan, Poland; iwona.kawacka@up.poznan.pl (I.K.); marcin.schmidt@up.poznan.pl (M.S.)

**Keywords:** CAMP test, food safety, species identification

## Abstract

*Listeria monocytogenes* is a foodborne pathogen that typically presents β-hemolytic activity. However, there are literature reports indicating that *L. monocytogenes* strains are sometimes nonhemolytic or their zones of hemolysis are perceivable only after removal of the colonies from the agar plate. Nonhemolytic *L. monocytogenes* are most commonly encountered in food products, but some have also been detected in clinical samples. Usually, atypical bacteria of this species belong to serotype 1/2a. Mutations of the *prfA* gene sequence are the most common reason for changed phenotype, and mutations of the *hly* gene are the second most common cause. There are also reports that the methodology used for detecting hemolysis may influence the results. Sheep or horse blood, although most commonly used in modern studies, may not allow for the production of clear hemolytic zones on blood agar, whereas other types of blood (guinea pig, rabbit, piglet, and human) are more suitable according to some studies. Furthermore, the standard blood agar plate technique is less sensitive than its modifications such as bilayer or top-layer (overlay) techniques. The microplate technique (employing erythrocyte suspensions) is probably the most informative when assessing listerial hemolysis and is the least susceptible to subjective interpretation.

## 1. Introduction

*Listeria monocytogenes* is a ubiquitous bacterium that is sporadically found in various food products, both processed and fresh or raw. It is also tolerant to high salt concentrations, low pH and low temperatures with the ability to replicate under refrigeration conditions. Once ingested with contaminated food, the bacteria can cause an illness, called listeriosis, with symptoms ranging from mild gastroenteritis to bacteremia, septicemia, meningitis, and abortions or stillbirths in the case of pregnant individuals [1,2]. Fully developed listeriosis has a mortality rate described as high, relatively higher compared to other foodborne illnesses [1,2,3,4]. In a prospective cohort study performed in France, called the MONALISA study, the 3 month mortality rate was estimated to be 46% for bacteraemia and 30% for neurolisteriosis cases [5]. However in other studies, the mortality rate is often lower, for example, it reached 16.8% among patients admitted to Spanish hospitals between 2001 and 2016 [6] or 18.3% among patients administrated to the hospital in a case-control study conducted between 2010 and 2019 in Japan [7]. European Union (EU) case fatality reached 15.6%, 13.6%, and 17.6% in 2017, 2018, and 2019, respectively, which makes listeriosis one of the most serious foodborne diseases under EU surveillance [8]. The EU notification rate of confirmed listeriosis cases was 0.46 cases per 100,000 population in 2019 [8], whereas the death rate from listeriosis was estimated to be approximately 0.13 patients per 100,000 population in a study performed in Spain [6].

β-Hemolysis, which is the ability to completely lyse red blood cells (erythrocytes) [9], is considered a species characteristic of *L. monocytogenes*. It is an important phenotypic criterion to differentiate *L. monocytogenes* from *L. innocua* [10,11,12,13,14]. Modern books and publications still state that *L. monocytogenes* is β-hemolytic without pointing to possible exceptions [4,15,16]. There are also many publications discussing the diverse aspects of *L. monocytogenes* and, in some of them, the lack of β-hemolysis presented by collected isolates would probably be considered an exclusive criterion, as authors confirmed species identification (apart from testing other traits) with β-hemolytic tests [17,18,19,20]. However, Lindbäck et al. (2011) pointed out that *L. monocytogenes* may easily be overlooked when the identification of *Listeria* species is based upon hemolysis, as there are some literature reports discussing atypical *L. monocytogenes* isolates that show not only weak but also complete lack of hemolysis [12,21], and there are atypical *L. innocua* isolates that exhibit hemolysis [22,23]. Furthermore, there are findings suggesting that isolates presenting hemolytic activity may be determined as non-hemolytic, because different methodologies have variable sensitivities [24]. The issue was also addressed many years earlier by authors who suggested that hemolysis of *Listeria* spp. is often weak or questionable, which as a consequence leads to subjective interpretation [25].

The aim of this review was to gather reports on atypical nonhemolytic *Listeria monocytogenes* strains in order to estimate the prevalence rate among diverse samples, present reasons underlying the atypical phenotype, and to summarize and organize reports about techniques used for assessing listerial hemolysis.

## 2. Hemolytic Phenotype of *L. monocytogenes*

The hemolytic activity of *L. monocytogenes* is determined by hemolysin, specifically listeriolysin O (LLO), which is a pore-forming protein (sometimes referred to as Hly) encoded by the *hly* gene. Upstream of *hly*, there is also a *prfA* gene that encodes the master virulence regulator PrfA, required for, apart from many other genes, *hly* expression [26,27,28]. In general, nonhemolytic *L. monocytogenes* strains were considered less pathogenic than typical *L. monocytogenes*, owing to the fact that production of LLO was proved to be essential for virulence of these bacteria, as a strict relation between hemolytic activity and virulence potential was found [29]. Moreover, many nonpathogenic isolates show weak or a lack of hemolysis [30,31]. Due to the fact of these reports, a classically hemolytic phenotype was considered to be a virulence marker of *L. monocytogenes* [32,33]. However, listeriolysin is not the only factor involved in virulence [31], and even though to our knowledge it is still the most important virulence agent [27], we now know that during infection *L. monocytogenes* can spread to distant organs even in the absence of LLO expression [27,28]. It was also reported that strains responsible for clinical cases do not always present high hemolytic activity [31], which indicates the importance of monitoring (e.g., in food industry) both hemolytic and nonhemolytic strains of *L. monocytogenes*.

Interestingly, although a diminished hemolytic phenotype may, to some extent, be related to hypovirulence, it does not necessarily indicate that *L. monocytogenes* strains presenting increased hemolytic activity are hypervirulent. In contrast, the self-limiting expression of virulence factors, in the case of *L. monocytogenes*, restricts host cell damage, which prolongs the intracellular life of the pathogen, thereby promoting a persistent infection state [28]. Prevention of excessive LLO-induced cell damage allows to avoid premature destruction of the replicative niche of the pathogen, which is an important aspect of the cytosolic phase of listerial infection [27]. For example, in one study, the authors analyzed *L. monocytogenes* strains in which the virulence potential was attenuated. Within the studied group, there was one strain with higher than wild-type hemolytic activity. That trait alone was a probable cause of virulence attenuation in the case of the isolate. According to authors, overexpression of the hemolysin gene causing excessive host cell membrane damage led to exposure of the bacterium to the extracellular milieu, which subsequently resulted in smaller plaque formation in the plaque assay and a virulence-attenuated phenotype in an animal infection model [34].

## 3. Nonhemolytic Phenotype of *L. monocytogenes*—Prevalence Rate

As mentioned earlier, *L. monocytogenes*, in general, is considered β-hemolytic, and its hemolytic activity is often used as a criterion to confirm species identification. However, there are findings indicating that nonhemolytic *L. monocytogenes* are also sporadically encountered, especially in samples from food and food-processing environments. Nonetheless, these bacteria were also found in clinical samples.

In a recent study, the hemolytic activity among *L. monocytogenes* was screened by [26]. The authors included 57,820 *L. monocytogenes* isolates, which strongly outnumbers every other report of that type made to date. The prevalence rate of nonhemolytic *L. monocytogenes* isolates according to that study was 0.1% [26]. On the other hand, there are also reports indicating that nonhemolytic *L. monocytogenes* isolates constitute more than 20% and up to 85.7% of all samples collected by other authors [10,21,35,36,37]; however, in some cases, there is the possibility that bacterial clones (well established in a facility or particular environmental niche) were collected in multiple samples or that the results were overestimated due to the small number of specimens included in the study. Summarized literature reports about nonhemolytic or weakly hemolytic *L. monocytogenes* are presented in Table 1.

Out of 57,820 *L. monocytogenes* samples from various sources (including food, clinical, veterinary, and environmental) collected between 1987 and 2008, 60 were identified as nonhemolytic. Most of the atypical isolates originated from food and food production environments (35 samples) and only three originated from human clinical cases, whereas the rest (22 samples) originated from unknown but nonhuman sources [26]. In another study, where 181 *L. monocytogenes* samples originating from human clinical, animal clinical, food, and environmental sources were included, the authors found four isolates that showed no hemolysis on blood agar plates. Three of them were collected from food sources and the fourth one was the strain type *L. monocytogenes* NCTC 10357 [40].

Examination of 26 *L. monocytogenes* strains originating from pork, slaughterhouses, markets, and human infections revealed that six isolates gave “weak positive” results in hemolysis assays; however, the authors did not precisely define the criteria for such a designation (other isolates gained either “positive” or “strong positive” results). All six isolates were collected at the same market (four from poultry and two from the floor) within an unspecified time frame by the authors. Furthermore, all isolates belonged to the 1/2a serovar [39], which altogether indicates that it potentially could be one persistent strain established in the facility. Similarly, in one fish processing plant, many nonhemolytic *L. monocytogenes* isolates were detected. Samples from equipment and final products were taken twice with a 6 month time span in between samplings. Out of 90 samples in total, 42 were positive for nonhemolytic *L. monocytogenes* (Yndestad and Hauge (2006), as cited in [21]). There was also a paper in which the authors reported that six out of seven *L. monocytogenes* isolates collected from milk products during routine sampling presented a nonhemolytic phenotype. All milk products were produced by the same manufacturer and all six strains belonged to the same serovar 1/2a [10].

In another report on a strain with a nonhemolytic phenotype that was presumptively persistent in an environmental niche related to marine samples (i.e., seawater, sediment and, shellfish), samples were taken from 18 sites located along an approximately 500 km of the Atlantic coast. Samples were collected over a period of two years. *L. monocytogenes* was present in 38 samples, and in eight cases the isolates were nonhemolytic. All eight isolates with that phenotype (although isolated within a significant time frame) shared the same pulsotypes via pulsed-field gel electrophoresis (PFGE) analysis and all belonged to serovar 1/2a [35].

From the abovementioned reports, it is apparent that nonhemolytic *L. monocytogenes* are most often found in food products and food-processing environments. One paper also reported that *L. monocytogenes* isolates lacking β-hemolysis were found in pet food. The isolate belonged to the 1/2a serotype [36]. On the other hand, there is also a report on *L. monocytogenes* from a diabetic dog with a urinary tract infection. The isolate showed “extremely weak” hemolysis, perceivable only after colonies were removed from the agar plat; the bacterium belonged to the 1/2a serovar [38].

Although most of the nonhemolytic *L. monocytogenes* strains belong to the same serovar 1/2a, it is worth mentioning that this is one of the serovars (along with 1/2b, 1/2c, and 4b) most frequently isolated in general [42,43,44].

## 4. Reasons Underlying Diminished Hemolysis

As mentioned earlier, the hemolytic phenotype of *L. monocytogenes* depends mostly on the *hly* and *prfA* genes. However, in the majority of cases, mutations within the *pfrA* gene are responsible for changes in phenotype, whereas *hly* mutations are detected less frequently.

In the largest study to date, performed by [26], which included 60 distinct nonhemolytic *L. monocytogenes* isolates, 56 of the strains had mutations in the central virulence regulator gene—*prfA*. Of those isolates, seven had PrfA protein with amino acid substitution and six had the protein enlarged, whereas in the rest (i.e., 43 strains) the protein was truncated. Two of the strains with mutated PrfA (both of them had a truncated version of the protein) presented mutations also in the *hly* gene, which subsequently resulted in Hly truncation. Three other nonhemolytic strains (without detected mutations in the *prfA* gene) showed mutations in the *hly* sequence: one had a truncated Hly protein and the other two had amino acid substitution in the Hly sequence). One strain presented complete loss of the *hly* gene and, interestingly, that isolate additionally showed no PrfA activity due to the missing *gshF* gene, critical for PrfA activation, even though the *prfA* sequence mutations within the gene of that strain have not been discovered. The research also revealed that atypical nonhemolytic strains are phylogenetically diverse, and the authors concluded that loss of hemolytic activity is caused by independent events across the *L. monocytogenes* population [26].

Mutation within the *prfA* gene was also reported in the case of *L. monocytogenes* strain isolated from a dog with a urinary tract infection, where nucleotide replacement led to substitution of glycine to aspartic acid at residue 145 within the critical helix-turn-helix motif of PrfA. The authors concluded that further research was necessary to determine whether that mutation was indeed responsible for the reduction in the hemolytic activity of that strain [38]. Similarly, whole genome sequencing of the nonhemolytic isolate originating from pet food revealed a single base pair deletion in the *prfA* gene, which led to truncation of the PrfA protein [36].

All six samples isolated from the same market with “weak positive” results of β-hemolysis tests had mutations in both the *prfA* and *hly* genes. In the *prfA* sequence, a deletion of five nucleotides led to truncation of the PrfA protein. The isolates also presented amino acids substitutions in the Hly protein sequence [39].

There is also a report suggesting that truncation of PrfA (and the subsequent lack of hemolytic activity) can be spontaneously reversible due to the fact of slipped-strand mispairing [21]. Two nonhemolytic *L. monocytogenes* strains were collected in the spring and autumn from the same fish processing plant in Norway and had indistinguishable PFGE patterns. When the atypical strains were injected intraperitoneally into mice, they caused death in 60% of the animals. Interestingly, from both the liver and spleen of all of the deceased mice, the authors recovered *L. monocytogenes* isolates that were both hemolytic and nonhemolytic. The PFGE patterns of the isolates of both phenotypes were indistinguishable from each other and also from the mother strain, which was originally injected into the mice. Sequencing of *prfA* genes was performed in both hemolytic and nonhemolytic strains. It revealed a duplication of seven base pairs in the nonhemolytic strain when compared to the hemolytic strain and control EGDe strain. The duplication changed a reading frame and resulted in a truncated PrfA protein, which led to a subsequent lack of hemolytic activity. The authors concluded that slipped-strand mispairing was a mechanism that resulted in excising the repeat and suggested that the aforementioned mechanism regulated phase-variable expression of virulence in *L. monocytogenes* [21]. Interestingly, a seven bp repeat was also observed in three other strains isolated in France ([31,45,46] as cited in [21]), which suggest that isolates with the 7 bp repeat mutation can potentially be spread worldwide.

On the other hand, there is a report of the spontaneous loss of hemolytic activity in *L. monocytogenes* ATCC 35152 that was originally hemolytic. On blood agar plates, the hemolytic and nonhemolytic colonies occurred in ratios of approximately 3:1 to 2:1 and had stable phenotypes when they were restreaked on fresh blood agar. Investigations in other laboratories supported that, indeed, *L. monocytogenes* ATCC 35152 had two phenotypes [47], further supported by a publication by other authors [48]. However, the molecular mechanism responsible for the switch from a hemolytic to a nonhemolytic phenotype has not been determined.

Apart from Hly and PrfA, it was also demonstrated that cold shock proteins (Csps) are important factors in terms of hemolysin expression and exhibiting a hemolytic phenotype. Although deletions of single *cps* genes did not influence hemolysis on blood agar plates, the *L. monocytogenes* EGDe strain lacking all three listerial Csps (Δ*cspABD*) caused diminished hemolysis on agar blood plates, which was further confirmed by analyses on bacterial supernatants, as the strain caused four-fold less hemolysis than the wild-type strain. Double deletion mutants harboring only either *cspA* (Δ*cspBD*) or *cspD* (Δ*cspAB*) caused less hemolysis than the parental wild-type strain, whereas mutants harboring only *cspB* (Δ*cspAD*) showed a hemolysis phenotype that was not significantly lower or even marginally better compared to the wild-type strain. According to the authors of the study, CspB seems to be the most significant for the hemolytic phenotype, as it is sufficient to maintain wild-type levels of LLO activity and gene expression [49].

## 5. Impact of Methodology on the Hemolytic Phenotype

Apart from molecular mechanisms responsible for atypical phenotype, there are also methodological details that may influence the results of hemolysis assessment. In general, hemolysis of *Listeria* spp. is often weak or questionable which, as a consequence, may lead to subjective interpretation of the results [25]. Moreover, different methodologies have variable sensitivity [24], and sometimes obtained results may appear elusive, and they are not always consistent within methodologies. For example, there is a report on *L. monocytogenes* strains that demonstrated variable phenotypes, sometimes appearing as hemolytic and sometimes as nonhemolytic when stabbed on a blood agar plate “with usually no discernible pattern emerging” [50].

There are many protocols designed and used for the assessment of hemolytic activity of *L. monocytogenes* and other *Listeria* species. However, most of these methodologies can be classified into five general types, which then vary in the details. The methodologies used for assessing listerial hemolysis are not systemized in the literature and sometimes referred to with different names. Herein, we aimed to classify the methodologies into groups and summarize the reports on their effectiveness.

### 5.1. Hemolysis Assays

#### 5.1.1. Blood Agar Technique

The most basic method is the blood agar technique, currently most often applied by authors who perform hemolysis assays of *Listeria* spp. [21,23,26,35,38,39,49,51,52]. Blood or blood cells are added to agar medium and, thus, blood is equally distributed on the agar plate. After solidification of the medium, bacteria are streaked on the agar surface, incubated, and screened for hemolytic zones. The incubation time is usually 24 h [26,47,49,50,51,53], sometimes prolonged to 48 h [21,26,47,50] at temperatures ranging from 35–37 °C [21,26,38,47,49,51,53]. Usually, Columbia blood agar plates are used [23,26,38,49], but in earlier studies, authors reported using Tryptic soy agar [30,50,53] or other media, e.g., heart infusion agar [47]. The concentration of defibrinated blood is usually 5% (*v*/*v*) in the agar medium [30,38,39,47,53,54].

Positive control strains *L. monocytogenes* CLIP 74910 [23,26] and *L. monocytogenes* 10403S [52] as well as negative control strains *L. innocua* CLIP 74915 [23,26] and *L. innocua* ATCC 33090 [52] were used in some studies.

The methodology allows for not only reading the results as positive or negative, but the authors were able to differentiate (e.g., strong, moderate, weak, or very weak) hemolysis based on the hemolytic zone size [39,55].

However, previous studies showed that it was sometimes necessary to remove bacterial colonies from the agar surface in order to see hemolytic zones that appeared only in the contact area [38,55], although one author, who also observed the phenomenon, decided to refer to those types of colonies as nonhemolytic as opposed to those giving clear zones of hemolysis around bacterial colonies [47]. To prevent achieving that type of equivocal results and to provide a clear-cut reading of hemolytic activity, a modification of the standard method was proposed. The methodology requires the use of an exceptionally thin layer of blood agar medium (8 mL) and inoculating the plate on a small area with a heavy cell mass. The incubation time is shorter than normal and lasts 6 h [56]. Utilization of a thinner layer of blood agar medium allowed for observation of a narrow zone of hemolysis around colonies of *L. monocytogenes*, which previously gave ambiguous results in hemolytic activity assays [56].

#### 5.1.2. CAMP Test

Another method to assess the hemolytic activity of *Listeria* spp. is to use the CAMP test, which similarly to the aforementioned technique, requires the use of blood agar plates. The test was based on the principle that hemolysis of *L. monocytogenes* is enhanced in the vicinity of *Staphylococcus aureus* and precisely its β toxin [13,57]. The name “CAMP test” finds its origin in the first letters of the original authors (Christie, Atkins, and Munch-Petersen) of the note about the phenomenon of *Staphylococcus* β toxin, which showed the ability to enhance or induce hemolysis around colonies of some *streptococci* isolates on blood agar plates [58,59].

Darling (1975) [59] proposed a standardization of the CAMP test procedure. He recommended using sheep blood agar plates on which β-toxin-producing *S. aureus* is streaked in a straight line across the center of the plate. Then, strains of the microorganisms to be tested are streaked in straight 2–3 cm lines at a right angle to the *S. aureus* streak but with caution not to touch the *Staphylococcus* line. Plates are incubated at 37 °C. Enhancement of the hemolysis of the tested isolate in the vicinity of *S. aureus* and the appearance of the “arrowhead” shape of hemolysis, pointing towards the *Staphylococcus* streak, indicate positive CAMP test results [59]. Currently, when *Listeria* isolates are tested, it is proposed to make two parallel streaks of *S. aureus* and additionally *Rhodococcus equi* with an approximately 3–4 cm space between the lines. Then, tested isolates are inoculated with a streak at a right angle to both previously made lines, but not touching them (leaving approximately a 1–2 mm space between streaks). *L. monocytogenes* hemolysis is enhanced by *S. aureus*, but *L. innocua* hemolysis is enhanced by *R. equi*, which allows for species differentiation [13,16]. There are also variations of the CAMP test, where commercially available discs soaked with *Staphylococcus* β toxin are used instead of the streaking bacterial culture of *S. aureus* [13,37].

Because of the enhancement of hemolysis exhibited by *L. monocytogenes*, a CAMP test with *S. aureus* can sometimes be used to resolve questionable hemolysis results [15]. However, the CAMP test may also generate false-positive and false-negative results due to the subjectivity of the interpretation [60] and provide ambiguous “plus–minus”-type reactions [37]. Some scientists proposed using other techniques (especially the microplate technique) as a reliable methodology to assess the hemolytic character of *Listeria* [60] or to change the type of blood to guinea pig which, according to authors, renders hemolysis-enhancing methods, such as the CAMP test, unnecessary [48].

#### 5.1.3. Top-Layer (Overlay) Technique

The top-layer technique, also referred to as the red blood cells top-layer technique [24] or overlay technique [61] has been reported as a more sensitive alternative for the standard blood agar technique [61]. This methodology was originally designed for detection of listerial hemolysis directly on selective plating media, which simplifies and rationalizes screening for *L. monocytogenes* colonies among those that grow on selective plating media [61,62].

In this technique, selective plating media (without blood addition) are streak inoculated with bacteria and incubated at 37 °C for 48 h. After incubation, plates are maintained at 4 °C for 2 h, and then a thin (8 mL) top layer of nonselective (BHI) medium with red blood cells is gently poured on the top of the base layer. Plates are then incubated again for 14 h at 30 °C and screened for hemolysis [61,62].

Other authors reported also using nonselective media as a base layer (namely, a brain heart infusion (BHI) agar or Columbia agar base, either with or without the addition of potassium tellurite) [24,32], increasing the top-layer volume up to 15 mL [32] and changing the conditions of second incubation to 24 h at room temperature [32].

There is also a variation of the top-layer technique where wells are made in the base layer using a drill bit, filter-sterilized bacterial culture supernatants are then introduced into the wells (in portions), and plates are stored to allow supernatant absorption. After a given time, the non-absorbed supernatant is removed, and a top-layer is added. Plates are then incubated and hemolysis is expressed as a diameter of the zones of hemolysis [32].

With the top-layer technique, *Listeria* gave much clearer hemolytic areas on every tested selective plating medium using sheep red blood cells compared to when sheep red blood cells or sheep blood were incorporated into the base medium as traditionally performed [61,62]. *Listeria* agar selective medium modified (LSAMM) was suggested to be the most suitable selective plating medium to be used with the overlay technique, as the hemolytic zones that were big, sharp, and easily recognizable [61,62].

When nonselective agar media were employed as a base layer, *L. monocytogenes* displayed bigger zones of hemolysis on BHI agar than on Columbia agar base when sheep blood was used [32], which was also confirmed in other study [24]. Hemolytic zones on both types of media with horse or human blood were comparable [24].

Similar to the standard blood agar technique, this method allows to estimate the hemolytic power of the isolates. The authors were able to differentiate (e.g., strong or moderate) hemolysis [24]. However it was sometimes necessary to remove the colony from the medium to perceive hemolysis that occurred only in a contact area [24].

#### 5.1.4. Bilayer Technique

The bilayer technique (sometimes referred to as the “blood agar” technique [24]), similarly to the top-layer technique, requires using plating agar media that consists of two layers: the base one without the addition of blood and the top one that contains red blood cells. The difference between the bilayer and top-layer methods is that in the bilayer technique, bacteria are inoculated on the top of the second layer.

The plate, as originally designed, consists of one layer (10 mL) of Columbia blood agar base (without blood) and a 5 mL top layer of the same agar supplemented with 5% horse blood [63]. Bacterial colonies are surface inoculated and incubated overnight (16–24 h) at 35 °C with 5% CO_2_ [63].

Modifications of the procedure proposed by other authors include using more medium (15 mL) in the first layer [12], using less (4.5 mL) [48] or more (8 mL) [12] medium in the second layer, employing other types of base media [12,24] as well as other types of blood (sheep, guinea pig, or human) [24,48], and performing aerobic incubation [12,24,48] at 37 °C [48] for up to 48 h [12,24,48].

According to authors of original methodology, β-hemolysis zones of *Listeria* are not perceivable on bacterial colony counters with that technique, but they can be seen by tilting the plate at an angle oblique to a fluorescent desk lamp [63]. However, other authors did not report using a fluorescent desk lamp to read the results [12,24,48].

According to the authors that compared nine different media, blood agar base no. 2 and Columbia blood agar base were the best choices for detecting the hemolytic activity of *L. monocytogenes* when employing a bilayer technique [12].

Similar to the standard blood agar technique, this method allows to estimate the hemolytic power of the isolates. The authors were able to differentiate (e.g., strong or moderate) hemolysis [24]; however, it was, in some cases, necessary to remove the colony from the medium to determine the hemolytic activity [48].

#### 5.1.5. Microplate Technique

The microplate technique (called also the microfuge-tube assay [37], microwell hemolysis test [48], or micro-technique [55]) was proposed as an alternative to other methods aiming to determinate listerial hemolytic activity. The methodology employs erythrocyte suspensions and eliminates interpretation difficulties that may be encountered when assessing hemolysis produced by *Listeria* spp. on blood agar plates [55]. It was originally used to determine the activity of purified *L. monocytogenes* hemolysin [64] and not the hemolytic activity of bacteria or bacterial cell supernatants, but other authors have employed the test for these purposes.

The technique is based on preparing serial dilutions (usually in a 96-well microtiter plate [24,31,45,49,50,55,65]) of bacterial cultures [24,34,55], bacterial supernatants [66,67,68], or bacterial filtrates [31,45,49,50,65] and then adding a standardized amount of blood or red blood cells suspensions to each dilution. Some authors use bacterial suspensions or bacterial cultures that have a standardized optical density (OD) before the hemolysis assessment [34,49,65,66]. Blood addition is followed by incubation, which is significantly shorter than in other methods, and lasts 30 min [69], 40 min [37,49], 45 min [24,66], 1 h [34,50,64], 3 h [31,45,65], 6–8 h [55], or 8 up to 10 h [67,68] depending on the author’s choice. The incubation temperature is 37 °C for the majority of protocols [24,31,34,45,49,55,64,65,66,67,68,69].

After incubation (and sometimes centrifugation of well contents [31,37,45,65,66]), hemolysis is measured. Usually, it is performed by visual scoring [34,50,55,64,67,68], but it can also be assessed with a spectrophotometer [49,65,66].

Hemolytic activity is usually expressed in complete hemolytic units (CHUs), which are defined as the reciprocal of the highest dilution at which complete hemolysis occurred [34,55,64,69]; however, some authors decide to also determine the minimal hemolysis unit (MHU), which is defined as the reciprocal of the highest dilution at which any hemolysis was detected [55,68]. There are also hemolytic units (HUs) based on the reciprocal of the highest dilution at which at least 50% hemolysis of the erythrocytes could be observed [31,45,50,66]. In the case of absorbance measurements, the results can be expressed as a percent of hemolysis in relation to a negative control which is set to 100% [49]. In one case, hemolytic units were expressed as a percentage of the hemolysis of the bacterial control strain (10403S) [34].

In some experiments based on this technique, the authors reported using additives that enhanced listerial hemolysis. Treating horse red blood cells with crude exosubstances of *S. aureus*, *R. equi*, *Pseudomonas fluorescens*, or *Acinetobacter calcoaceticus* prior the microplate assay led to enhancement of the hemolytic activity of *L. monocytogenes* isolates, which was expressed in an increase of CHUs or MHUs or both of the parameters [55]. Similarly, potassium tellurite in a range of concentrations from 0.004% to 0.02% added to the media enhanced hemolysis presented by *L. monocytogenes* cultures [32], as well as charcoal-treated broth utilization (compared to untreated medium) led to approximately 10 times higher hemolytic activity of *L. monocytogenes* strains [66].

Some authors reported using positive and negative controls with this technique. *L. innocua* ATCC 33090 [37], phosphate buffer saline used instead of bacterial supernatant [49], or uninoculated medium [65] served as negative controls. With *L. monocytogenes* NCTC 7973 [37], sodium dodecyl sulfate (0.1%) used instead of bacterial culture [65] or blood samples fully hemolyzed by 0.05% Triton X [34] served as positive controls.

The methodology also has a simplified version that does not require preparation of serial dilutions. Bacterial suspensions are directly placed in one well or tube and a standardized amount of blood is added. The degree of hemolysis is assessed after incubation. It allows for the differentiation (e.g., complete, strong, and moderate) hemolysis or negative results [24,55].

This methodology provides unequivocal results, which allow to clearly differentiate between weak and strong hemolytic strains [55], as the results can be presented in comparable units. This methodology provided clear-cut readings in cases where the CAMP test gave ambiguous results [37], and even the simplified version of the microplate technique was assessed as a reliable, simple, and fairly rapid method for clearly differentiating between hemolytic and nonhemolytic *Listeria* strains [24].

### 5.2. Blood Type Impact

The origin of red blood cells (RBCs) may have an impact on the results of hemolytic assessments. Literature reports are not consistent with the findings regarding which blood type is the best for those types of studies. The authors of more modern experiments usually use either horse [26,51,70] or sheep [38,39,52] RBCs when testing the hemolysis of bacteria from the *Listeria* genus; however, some authors decide to use alternatives such as bovine [49] or human [65] blood.

In one study, *L. monocytogenes* 15313 gave clear hemolytic zones when human (types A+, B−, AB−, O+), rabbit, piglet, and chicken blood types were used; on bovine blood, it gave a weak response, whereas no hemolytic reaction was observed with sheep or horse blood [71]. The lack of β-hemolysis of these strains on agar containing sheep or horse blood was also reported earlier by other authors [72]. In another report, *L. monocytogenes* isolates originating from food produced clear zones of hemolysis on guinea pig blood agar, while lytic zones on sheep blood agar did not extend beyond the edge of the colonies and could only be confirmed after removal of the colonies. According to the authors, zones of β-hemolysis on guinea pig blood are clearer than on other (i.e., cow, horse, sheep, and rabbit) types of tested blood [48].

In other paper comparison of RBCs types led to conclusion that hemolytic activity of analyzed isolates was stronger in the presence of RBCs from sheep or guinea pig compared to horse and human, where lytic zones were very small or even questionable [24]. Blood type had impact on the results only when some techniques were used (namely bilayer technique or top-layer technique with selective media), whereas in other (microplate technique or top-layer technique with nonselective media) blood type had little or no impact [24].

Abovementioned findings are not in agreement with the paper in which *L. monocytogenes* strain SO93 had no hemolytic activity when sheep RBCs were used, but exhibit hemolysis on horse RBCs [45]. Similarly 24 *L. monocytogenes* strains were found to present stronger hemolytic activity on media containing horse blood than on those containing sheep blood [12].

## 6. Summary and Concluding Remarks

Although *L. monocytogenes* is considered β-hemolytic microorganism, there are reports about isolates of atypical phenotype. The largest study performed to date determined that prevalence rate of nonhemolytic *L. monocytogenes* reaches approximately 0.1% [26]. Nonhemolytic isolates are encountered in food and food processing environments in majority of cases, however some clinical isolates also presented diminished hemolysis. Usually weak or lack of hemolysis is caused by mutations within central virulence regulator *prfA* or hemolysin *hly* genes. There are however still some unexplained reports regarding nonhemolytic phenotype, e.g., in case of *L. monocytogenes* ATCC 35152 strain which spontaneously lost hemolytic activity [47].

Hemolysis of *L. monocytogenes* is sometimes difficult to assess and many authors have dedicated their time to eliminating ambiguous results that were obtained during the tests. Although many publications present inconsistent findings regarding, for example, medium that enables the easiest interpretation of the results or the most accurate blood type, there are many valuable protocols to be considered when testing the hemolytic abilities of *Listeria* spp. isolates. The microplate technique appears to be the least susceptible to subjective interpretation and provides results expressed in units that can be easily compared within specimens.

Due to the reports regarding sporadic nonhemolytic *L. monocytogenes* isolates (as well as sporadic hemolytic *L. innocua*) and considering the limitations of the methodologies available for assessing the listerial hemolytic phenotype, we conclude that species identification based on hemolytic abilities should be performed with exceptional caution in the case of the *Listeria* genus. Potential overlooking of *L. monocytogenes* isolates may, for example, lead to life-threatening infections in the case of food samples, as there are reports about weakly hemolytic isolates that caused clinical infections, and there are publications addressing the issue of isolates that switch their phenotype. However, rejection of nonhemolytic specimens from scientific studies addressing diverse aspects of *L. monocytogenes* is also very concerning, and it may be a reason for the possible underrepresentation of the isolates of that type in many publications.

## Figures and Tables

**Table 1:** Nonhemolytic or weakly hemolytic *L. monocytogenes* strains reported in the literature.

Number of *L. monocytogenes* Isolates in the Study	Origin of the Isolates in the Study	Number of Nonhemolytic Isolates (%)	Number of Isolates with Weak Hemolysis (%)	Reference
57,820	Food, clinical, veterinary, environmental, and other	60 (0.1%)	N/A ^1^	[26]
1	Dog urinary tract infection	N/A	1 (100%)	[38]
3	Pet food	1 (33.3%)	N/A	[36]
26	Pork, slaughterhouses, markets, and human infections	N/A	6 (23.1%)	[39]
Not specified ^2^	Equipment and products from one plant producing smoked salmon	42 (–)	N/A	Yndestad and Hauge (2006), as cited in [21]
38	Seawater, sediment, and shellfish	8 (21.1%)	N/A	[35]
7	Milk products from one manufacturer	6 (85.7%)	N/A	[10]
181	Human clinical, animal clinical, food, and environment	4 (2.2%)	N/A	[40]
12	Smoked fish	1 (8.3%)	N/A	[41]
27 ^3^	Meat and poultry or obtained from a culture collection	12 (44.4%)	1 (3.7%)	[37]

^1^ N/A—not applied, isolates of that type were not discriminated in the reference; ^2^ 90 samples were taken, but the precise number of collected *L. monocytogenes* isolates was not specified; ^3^ only isolates identified as *L. monocytogenes* with both an API system and an ACCU-Probe were included.

## Data Availability

Not applicable.
